# Women's Interest in Midwifery Continuity of Care During and After Pregnancy and Childbirth in Sweden: “As a Matter of Course”

**DOI:** 10.1111/jmwh.70035

**Published:** 2025-09-27

**Authors:** Hanna Fahlbeck, Ingegerd Hildingsson, Birgitta Larsson, Margareta Johansson

**Affiliations:** ^1^ Department of Women's and Children's Health Uppsala University Uppsala Sweden; ^2^ Department of Women's Health Uppsala University Hospital Uppsala Sweden; ^3^ Department of Nursing Mid Sweden University Sundsvall Sweden; ^4^ Department of Health Promoting Science Sophiahemmet University Stockholm Sweden

**Keywords:** care model, childbirth, continuity of care, midwifery, pregnancy, woman

## Abstract

**Introduction:**

The midwifery continuity of care model is well‐established internationally, but it is rarely offered in Sweden. Pregnant women's interest in midwifery continuity of care has not been investigated in recent years. This study aimed to investigate the interest of pregnant women and new mothers in Sweden regarding midwifery continuity of care and to identify factors associated with this interest.

**Methods:**

A national longitudinal digital questionnaire was conducted to collect background information, pregnancy‐related variables, and childbirth‐related variables, as well as to measure interest in midwifery continuity of care among women in Sweden. Odds ratios with 95% CIs and logistic regression analyses were used.

**Results:**

Of 1697 women who responded, 68.1% expressed a strong interest in midwifery continuity of care during pregnancy, and 74.2% during postpartum. Fear of childbirth was associated with a higher interest in midwifery continuity of care during pregnancy (adjusted odds ratio [aOR] 1.75; 95% CI, 1.34‐2.27). Women who had mixed or negative experiences with the care they received were also more likely to be interested in the model (aOR, 2.33; 95% CI, 1.43‐3.97).

**Discussion:**

Pregnant women and new mothers in Sweden show a high level of interest in midwifery continuity of care. However, current maternity services do not adequately meet these preferences, indicating a need to scale up continuity of care models, particularly for women who experience fear of childbirth and dissatisfaction with their care. Therefore, antenatal, intrapartum, and postpartum care in Sweden should be enhanced to better align with the needs and preferences of pregnant women and new mothers.

## INTRODUCTION

Midwifery continuity of care (MCoC) is a care model in which a woman is cared for by the same midwife, or a small group of midwives, throughout pregnancy, labor, birth, and early parenting. This approach fosters a trusting relationship between the woman and her midwife.^1^ Although MCoC is common internationaly,^2^ it is rare in Sweden.^3^ A recent systematic review^2^ comparing MCoC with other care models during pregnancy and childbirth highlights several benefits. Women receiving MCoC were more likely to have a spontaneous vaginal birth and less likely to undergo a cesarean or instrumental vaginal birth. They also reported higher satisfaction with their care and more positive experiences during pregnancy, labor, and the postpartum period. Additionally, cost‐saving effects were observed in both antenatal and intrapartum care.^2^ The World Health Organization (WHO) recommends MCoC for a positive pregnancy^1^ and childbirth^4^ experience.

**QUICK POINTS**

✦ The interest in midwifery continuity of care among pregnant women and new mothers in Sweden is high.
✦ Fear of childbirth and dissatisfaction with care are factors associated with such interest.
✦ Maternity services and childbirth care in Sweden should be enhanced to better align with the needs and preferences of pregnant women and new mothers.

Extensive research has investigated women's experiences with MCoC in countries where the model is implemented, showing overwhelmingly positive results.^5^, ^6^, ^7^, ^8^ Women in MCoC models report high levels of satisfaction with their care^9^, ^10^, ^11^ and perceive the quality of care as high.^12^ The relationship between the woman and her midwife is often described as fundamental and crucial.^8^ In a Swedish qualitative study, women expressed feeling safe and secure within a midwifery continuity model.^13^

In Sweden, midwives provide care for women throughout the entire reproductive life span. All midwives are trained to support women from menarche to menopause, including contraceptive and abortion counseling, as well as performing Pap tests. They serve as the primary health care providers for pregnant and birthing women, as well as for new mothers, and are autonomously responsible for the care of healthy women with uncomplicated pregnancies and births. Midwives work in close collaboration with obstetricians and other health care professionals and manage pregnancies and childbirths, including when complications arise. Midwives attend all births in Sweden, except in cases in which the birth occurs unexpectedly outside a health care facility or when the mother has chosen to give birth unassisted. In the Swedish maternity system, a pregnant woman is typically assigned to a specific midwife, ensuring a high level of continuity during pregnancy. On average, women have 9 antenatal visits at the primary health care clinic, and 63% of pregnant women meet a maximum of 2 midwives during this period.^14^ However, care during labor, birth, and the early parenting period is often fragmented, with women and their families encountering multiple unfamiliar midwives and other health care professionals in labor and postpartum hospital clinics. The majority of new mothers in Sweden return to their antenatal midwife for a routine appointment check‐up, often between 8 and 12 weeks postpartum.^14^ Only 1 out of Sweden's 21 counties has implemented a MCoC model^3^, and home births account for just a fraction of a percentage of all births in the country.^15^

Previous research indicates that around 50% of pregnant women in Sweden would like to know the midwife who assists them during childbirth.^16^ In recent years, pregnancy and childbirth have received increased attention in Swedish media, and initiatives to improve MCoC have been encouraged and requested by different stakeholders.^17^, ^18^ Therefore, this study aimed to investigate the interest of pregnant women and new mothers in Sweden regarding MCoC and to identify factors associated with this interest.

## METHODS

### Study Design

A national questionnaire with a prospective longitudinal design was used. This article adheres to the Strengthening the Reporting of Observational Studies in Epidemiology (STROBE) guidelines to ensure complete and transparent reporting of this observational study (Supporting Information: Appendix S1).

### Data Collection

Data collection initially took place in 7 counties without an MCoC model and encompassing 10 clinics nationwide that performed ultrasound imaging during midpregnancy (ie, between pregnancy weeks 17‐19). Most of these clinics were tertiary care centers, with 2 being university clinics. In 2023, the annual number of births at these clinics ranged from approximately 400 to 3700. Due to the extended duration of data collection, the research team broadened recruitment by disseminating information about the study and how to participate via social media platforms (eg, Instagram, Facebook) and pregnancy mobile applications (eg, Mom2B, Preggers, One Million Babies).

After completing the ultrasound examination, midwives provided women with a pamphlet containing study information and a QR code linking to the questionnaire. The same information was disseminated on social media platforms and mobile applications with a direct link to the questionnaire. This was accessible from December 1, 2023, to April 2, 2024. The first questionnaire was completed during pregnancy between weeks 8 and 42, with most responses collected between weeks 14 and 27 (45.9%). The questionnaire inquired if women wished to partake in a follow‐up questionnaire postpartum. If affirmative, women registered their email addresses and received an invitation link with up to 2 reminders. The postpartum questionnaire was completed 1 to 10 months postpartum, with the vast majority (97.6%) answering 1 to 2 months postpartum.

### Participants

Inclusion criteria required participants to be pregnant and comprehend Swedish.

### Measures

The main outcome variable, interest in the model, was rated on a 4‐point Likert‐type scale ranging from “very interested” to “completely uninterested” (“very interested,” “interested,” “uninterested,” “completely uninterested”). In the statistical analyses, the variable was dichotomized into “very interested” and “less interested,” with all other response options grouped under the latter category.

Background variables collected during pregnancy were age (20‐24, 25‐34, 35‐47 years), country of birth (Sweden, not Sweden), having a partner (no, yes), education level (9‐year compulsory school, high school, college or university), previous birth (no, yes), and gestational week (8‐14, 15‐27, 27‐42). Participants were also asked if they were familiar with MCoC prior to the study and were provided with a definition of MCoC (“with the concept ‘midwife‐led continuity model’ we mean a care model where one midwife or a small group of midwives cares for you and your family during pregnancy, birth, and the early period after the baby is born”). In addition, fear of birth was assessed during pregnancy using the Fear of Birth Scale (FOBS),^19^ which is a validated, brief and effective instrument for measuring fear of childbirth.^20^, ^21^, ^22^ Participants were asked, “How do you feel right now about the approaching birth?” and rated their fear on a scale from 0 (no fear) to 100 (strong fear), and their anxiety from 0 (calm) to 100 (worried), using a 100‐point visual analog scale (VAS), respectively. The final score was calculated as the average of the 2 ratings. Scores of 60 or higher were classified as indicating fear of childbirth, whereas scores below 60 were considered to indicate no fear.^19^

The follow‐up questionnaire included variables related to the birth, such as mode of birth (spontaneous vaginal, instrumental vaginal, or cesarean birth including both planned and emergency cesarean births). Additional variables included childbirth experience and satisfaction with care. To measure childbirth experience, participants were asked to place a marker on a VAS ranging from 0 (“very poor experience”) to 10 (“very good experience”). Based on clinical practice, a childbirth experience rated as less than positive was defined as a score of 4 or lower, which in many Swedish health care regions qualifies women to be offered supportive conversations or a postpartum follow‐up at the birth clinic.^23^ Satisfaction with care during childbirth was measured across 3 dimensions: satisfaction with emotional care, satisfaction with physical care, and overall assessment of care provided during childbirth. Each dimension was assessed using a 5‐point Likert‐type scale and subsequently dichotomized into “satisfied,” which included responses of “very satisfied” and “satisfied,” and “less than satisfied,” which included “neither satisfied nor dissatisfied,” “dissatisfied,” and “very dissatisfied.” The 2 questionnaires also included prompts for free‐text responses, such as “Would you like to comment?” or “Would you like to add anything?” to elaborate or provide additional comments on their answers.

### Data Analysis

Background characteristics were analyzed descriptively by calculating frequencies and proportions. To explore factors associated with interest in MCoC, crude odds ratios (ORs) and adjusted ORs (aORs) with 95% CIs were calculated for background, pregnancy, and birth‐related variables. Differences in interest in MCoC were analyzed using the McNemar test. Background variables that were statistically significant were used for adjustment in the final step of the analysis. Data collection and management were carried out using REDCap, hosted at Uppsala University. REDCap is a web‐based, secure platform designed to facilitate data capture for research studies. It features an intuitive interface for validated data entry, automated export functions for transferring data to common statistical software, and capabilities for integrating data with external sources.^24^ Data were analyzed using IBM SPSS version 28. The free‐text responses were read repeatedly by all authors to gain a naïve understanding of their content. Subsequently, the first author counted the responses and integrated them into the quantitative description of the women's background characteristics during pregnancy and childbirth and their interest in MCoC. All authors agreed with the integration of the written notes.

### Ethics

The study was approved by the Ethics committee, DNR 2021‐01941, and DNR 2023‐03995‐02, and conducted in accordance with the Code of Ethics of the World Medical Association.^25^ Participation was not expected to involve any risks. In case of questions or the need to provide feedback, contact information for the responsible researchers was provided. No one outside the research group had access to the answers. Participation was voluntary, as indicated in the written information and at the questionnaire's commencement. The initial statement clarified that filling out the questionnaire constituted consent to participate. Participants were informed of their right to withdraw at any time, without providing a reason.

## RESULTS

### Background Characteristics During Pregnancy

The section of the questionnaire addressing background variables was completed by 1789 women during pregnancy, with a mean age of 32.2 years. Almost all women had a partner (98.3%) and were born in Sweden (92.5%), as shown in Table 1. Among those born outside Sweden, 50 different nationalities were represented. Participants came from all 21 health care counties in Sweden, with the majority residing in the health care region Stockholm‐Gotland (30%). A slight majority of the women (55.1%) had previously given birth.

**Table 1:** Participant Characteristics During Pregnancy

Characteristics	N = 1789 n (%)^a^
**Age group, y**	
20‐24	46 (2.9)
25‐34	1081 (69.1)
35‐47	438 (28.0)
**Partnership status**	
Has a partner	1762 (98.3)
Does not have a partner	30 (1.7)
**Country of birth**	
Sweden	1633 (92.5)
Other country	133 (7.5)
**Education level**	
Compulsory school 9 y or less	23 (1.3)
Secondary school, 12 y of education	369 (20.5)
University education	1407 (78.2)
**Health care region**	
Northern region	216 (12.1)
Mid Sweden region	391 (21.8)
Stockholm and Gotland	549 (30.7)
Southeast region	90 (5)
West region	296 (16.5)
South region	249 (13.9)
**Living in a health care region where MCoC is implemented**	
Yes	549 (30.7)
No	1250 (69.3)
**Previously given birth**	
Yes	990 (55.1)
No	808 (44.9)
**Fear of Birth Scale**	
<60	1342 (76.8)
≥60 (fear)	405 (23.2)

Abbreviation: MCoC, midwifery continuity of care.

a Numbers might not add up to 100 % due to missing values.

During pregnancy, a total of 1388 women (82.0%) responded that they wanted to participate in the follow‐up questionnaire postpartum. Women who were not interested in the postpartum questionnaire had a lower education level (*P* < .001) but did not differ from the original sample in other characteristics (eg, partnership status, parity, age, or health care region). Most neonates were born full term (95.7%), spontaneous vaginal (77.4%), and in a labor ward (95.8%). Explanations for births outside the hospital included planned home birth (n = 27), unplanned home birth (n = 5), unknown (n = 3), and planned free birth (n = 1). The mean score for the childbirth experience was VAS 7.15. Most women were satisfied with their care across all 3 dimensions: emotional, physical, and overall, as presented in Table 2.

**Table 2:** Childbirth and Postpartum Variables

Characteristics and Outcomes	n = 1042 n (%)^a^
**Mode of birth, latest pregnancy**	
Spontaneous vaginal birth	807 (77.4)
Instrumental vaginal birth	53 (5.1)
Planned cesarean birth	62 (6.0)
Emergency cesarean birth	120 (11.5)
**Childbirth experience (VAS)** ^b^	
≤4 (less than positive)	158 (15.5)
5‐7 (positive)	303 (29.7)
8‐10 (very positive)	556 (54.7)
**Satisfaction with emotional care provided during childbirth**	
Very satisfied	522 (50.4)
Satisfied	296 (28.6)
Neither satisfied nor dissatisfied	131 (12.6)
Dissatisfied	65 (6.3)
Very dissatisfied	22 (2.1)
**Satisfaction with physical care provided during childbirth**	
Very satisfied	554 (53.1)
Satisfied	337 (32.3)
Neither satisfied nor dissatisfied	92 (9.6)
Dissatisfied	47 (4.5)
Very dissatisfied	9 (0.9)
**Overall assessment of care provided during childbirth**	
Very satisfied	522 (50.2)
Satisfied	368 (35.4)
Neither satisfied nor dissatisfied	86 (8.3)
Dissatisfied	53 (5.1)
Very dissatisfied	11 (1.1)

a Numbers might not add up to 100% due to missing values.

b VAS = visual analog scale, from 0 = very negative to 10 = very good.

### Interest in MCoC

Of all the respondents interested in MCoC during pregnancy (n = 1697), 68.1% (n = 1155) reported being very interested, and 27.2% (n = 461) were interested. A small proportion, 4.5% (n = 76), showed less interest, and only 0.3% (n = 5) were completely uninterested. This strong interest increased after childbirth, when 1036 women responded to the same question: 74.2% (n = 769) reported being very interested, and 20.4% (n = 212) were interested in the model. The same proportion of women, 4.5% (n = 47), showed less interest, whereas 0.9% (n = 9) were completely uninterested. Of the women who were very interested in MCoC during pregnancy (n = 723), 86.7% remained very interested postpartum. Among the women who were less interested during pregnancy (n = 313), 45% became very interested postpartum. The increase in women being very interested postpartum was statistically significant (*P* < .004).

During pregnancy, 27% (n = 487) of the women who showed interest in MCoC (“very interested” or “interested”) provided comments. The comments primarily focused on the desire to feel safe and secure (n = 220), followed by the wish to build a relationship with their midwife or midwives (n = 93). In some cases (n = 40), women stated that the model should be “a matter of course.” Women also expressed positive attitudes toward MCoC without further specification (n = 28). They also mentioned “positive outcomes” in general as something they associated with the model (n = 21). Some women explained that they lived in areas where MCoC was not available (n = 14) and therefore did not consider it an option. The importance of personal chemistry was also highlighted (n = 13). For 47% (n = 809) of the pregnant women, the concept of MCoC was new.

Of those showing less interest in MCoC during pregnancy (“less interested” or “very uninterested”), 40% (n = 32) left a comment. The most common responses included expressing that they were not in the right target group (n = 7), a wish not to know or ever meet the midwife assisting during birth (n = 6), or highlighting the importance of personal chemistry (and the risk of being assigned a midwife they did not like) (n = 5), prioritizing the skills of a midwife with rigorous experience in labor care (n = 4), having faith in every midwife (independent of the relationship) (n = 3), doubting the logistical planning and feasibility of the model (n = 3), planning not to give birth vaginally (n = 2), or sharing a previous negative experience with MCoC (n = 2).

### Factors Associated With Interest in MCoC

We compared women based on their interest in MCoC (Table 3). The following sociodemographic factors were associated with strong interest in MCoC during pregnancy: having a university or college level education (OR, 1.69; CI, 1.32‐2.15) and living in a region where MCoC is implemented (OR, 1.30; CI, 1.04‐1.63). Fear of childbirth was also associated with a strong interest in MCoC, and this association remained statistically significant after adjusting for relevant background variables (aOR, 1.86; CI, 1.37‐2.35). Being in the youngest age group (20‐24 years) was associated with less interest in MCoC (OR, 0.43; CI, 0.23‐0.83). No statistically significant associations were found with partnership status, country of birth, or parity.

**Table 3:** Background Variables by Interest in Midwifery Continuity of Care

Characteristics	Very Interested in MCoC n = 1155 n (%)^a^	Less Interested in MCoC n = 542 n (%)^a^	OR (95 % CI)	*P* value
**Age, y**				
20‐24	20 (2.1)	21 (5)	0.43 (0.23‐0.83)	.012
25‐34	668 (70.7)	280 (66.4)	1.08 (0.84‐1.40)	.53
35‐47	266 (27.9)	121 (28.7)	1 [Reference]	
**Civil status**				
Has a partner	1124 (98.0)	530 (99.1)	1 [Reference]	
Has no partner	23 (2.0)	5 (0.9)	2.17 (0.82‐5.75)	.12
**Country of birth**				
Sweden	842 (92.4)	383 (91.6)	1 [Reference]	
Other country	69 (7.6)	31 (5.9)	0.9 (0.59‐1.37)	.615
**Education level**				
High school or lower	207 (18.0)	146 (27.1)	1 [Reference]	
University education	942 (82.0)	393 (72.9)	1.69 (1.32‐2.15)	<.001
**Living in a health care region where MCoC is implemented**				
Yes	370 (32.3)	143 (26.6)	1 [Reference]	
No	777 (67.7)	394 (73.4)	1.30 (1.04‐1.63)	.023
**Parity status**				
Multiparous	649 (56.3)	291 (54.1)	1 [Reference]	
Primiparous	504 (43.7)	247 (45.9)	1.09 (0.89‐1.34)	.4
**Fear of Birth** **Scale**				
<60	820 (73.3)	441 (83.1)	1 [Reference]	
≥60 (fear)	292 (26.3)	90 (16.9)	1.75 (1.34‐2.27)	<.001

Abbreviations: MCoC, midwifery continuity of care; OR, odds ratio.

a Numbers might not add up to 100 % due to missing values.

When assessed postpartum, interest in MCoC was associated with satisfaction with care (Table 4). Women who were less than satisfied or had a mixed experience showed greater interest in MCoC. This trend was evident across all dimensions; emotional (aOR, 1.68; CI 1.15‐2.45), physical (aOR, 1.56; CI, 1.01‐2.4), and overall assessment (aOR, 2.34; CI, 1.34‐3.78).

**Table 4:** Pregnancy and Childbirth Outcomes by Interest in Midwifery Continuity of Care Postpartum

Characteristics and Outcomes	Very Interested in MCoC n = 769 n (%)^a^	Less Interested in MCoC n = 268 n (%)^a^	Crude Odds Ratio (95% CI)	Adjusted^b^ Odds Ratio (95% CI)
**Birth in gestational wk**				
24‐31	5 (0.6)	1 (0.4)	1.51 (0.17‐13.1)	1.6 (0.18‐14.1)
32‐36	26 (3.5)	11 (4.5)	0.68 (0.33‐1.5)	0.49 (0.36‐1.64)
37‐40	447 (59.7)	166 (63.4)	0.86 (0.62‐1.13)	0.86 (0.63‐1.17)
41‐42	271 (36.2)	84 (32.1)	1 [Reference]	1 [Reference]
**Mode of birth**				
Spontaneous vaginal birth	603 (78.6)	198 (73.9)	1.36 (0.95‐1.94)	1.34 (0.93‐1.91)
Instrumental vaginal birth	39 (5.1)	14 (5.2)	1.25 (0.63‐2.48)	1.21 (0.61‐2.42)
Cesarean birth	125 (16.3)	56 (20.9)	1 [Reference]	1 [Reference]
**Childbirth experience (VAS)** ^c^				
5‐10	635 (84.3)	218 (84.8)	1 [Reference]	1 [Reference]
≤4	118 (15.7)	39 (15.2)	1.04 (0.70‐1.53)	1.07 (0.72‐1.59)
**Satisfaction with emotional care provided during childbirth**				
Satisfied	558 (77.1)	226 (85.0)	1 [Reference]	1 [Reference]
Less than satisfied	175 (22.9)	40 (15.0)	1.68 (1.15‐2.45)	1.68 (1.15‐2.46)
**Satisfaction with physical care provided during childbirth**				
Satisfied	646 (84.1)	239 (89.2)	1 [Reference]	1 [Reference]
Less than satisfied	122 (15.9)	29 (10.8)	1.56 (1.01‐2.4)	1.56 (1.01‐2.41)
**Overall assessment of care provided during childbirth**				
Satisfied	639 (83.4)	246 (92.1)	1 [Reference]	1 [Reference]
Less than satisfied	127 (16.6)	21 (7.9)	2.34 (1.43‐3.78)	2.33 (1.43‐3.80)

Abbreviations: MCoC, midwifery continuity of care; VAS, visual analog scale.

a Numbers might not add up to 100% due to missing values.

b Adjusted for age, education level, living in a health care region where MCoC is implemented.

c From 0 (very negative) to 10 (very good).

## DISCUSSION

The main findings of this study are that pregnant women in Sweden showed significant interest in MCoC and that the interest increased after childbirth. Women perceived several potential advantages in participating in an MCoC model. Women who feared childbirth and those who were less satisfied with the care received during labor and birth were more likely to express a strong interest in MCoC.

The current study showed that 68.1% of women were interested in MCoC during pregnancy, and 74.2% were interested after childbirth. Significant associations were found with only a few sociodemographic variables, which is surprising given previous research linking interest in building a relationship with one's midwife to maternal age, partnership status, and parity.^16^ This difference could possibly be explained by the fact that interest in MCoC in the present study was comparatively higher. Despite previous research exploring women's interest in knowing their midwife throughout the childbearing continuum, only one Swedish region has established MCoC as an alternative to the standard fragmented care currently provided.^3^ According to the findings of this study, the Swedish health care system does not adequately address women's interest in MCoC during pregnancy, childbirth, and the early parenting period. Although women in Sweden generally express satisfaction with the care received during pregnancy, labor, and birth and rate antenatal clinics and labor wards highly, only 49% feel that their needs during childbirth and the postpartum period are fully met by the health care services.^14^ An even smaller proportion, 36%, report that health care services fully meet their needs throughout pregnancy, labor, birth, and the postpartum period.^14^ These ratings from the Swedish Pregnancy Register highlight significant gaps in meeting the needs of pregnant women, as recognized by health authorities.^26^

Swedish health care decision‐makers and providers must enhance the availability of care models to better align with women's preferences for different models of care throughout the childbirth continuum. Additionally, the Swedish National Board of Health and Welfare calls for increased continuity in postpartum care.^26^ Although expanding the midwifery model of care has been recommended to improve maternal and neonatal outcomes in the United States,^27^ the significantly lower number of midwives in the United States compared with other high‐income countries^28^ underscores the urgent need to strengthen the midwifery workforce to support the implementation of MCoC. In its recently published global position paper, the WHO encourages a reorientation of health care systems where the risk‐oriented, fragmented model of care is replaced by inclusive, person‐centered, respectful midwife‐led care whenever possible.^29^ Although MCoC is not the sole solution to meeting women's preferences, introducing alternative care models could be a viable approach to improving maternity services in Sweden.

### Fear of Childbirth

This study found that fear of childbirth was associated with a strong interest in MCoC. Fear of childbirth is a complex phenomenon, associated with factors such as lack of social support, mental health during pregnancy, and experiences during labor and birth.^30^, ^31^ Although the midwife‐woman relationship in an MCoC model is not intended to be overly personal, midwives should provide professional social and emotional support, based on the woman's preferences and needs. Previous studies have demonstrated positive outcomes of the MCoC model for women who fear childbirth, suggesting it as a valuable intervention.^32^, ^33^, ^34^ Adequate treatment for fear of childbirth is crucial, as research shows that fear can affect women for several years, influencing the intervals between pregnancies, preferences for pain relief during labor, and the duration and mode of birth.^27^ Severe fear of childbirth can lead to psychosocial distress, interfering with daily life, daily functioning, and bonding with the fetus.^35^, ^36^

A possible explanation for the finding of higher interest in MCoC among women with fear of childbirth is the opportunity for mutual preparation. In an MCoC model, women can prepare and plan for labor with the midwives who will assist them during birth. This contrasts with creating a plan with someone who will not be present at the birth, only to be assisted by an unfamiliar midwife, who tries to follow a plan made by someone else. Many midwives in antenatal clinics have not recently, or ever, worked in the labor ward. This can impact the planning and arrangements made for labor and birth if the antenatal care midwife provides counseling. Most regions have specialized counseling clinics for women with fear of childbirth, in which midwives are trained in consultative conversations.^37^ However, in an MCoC model, the midwife attending the birth could also provide counseling, allowing for more personalized preparations and plans. This planning is available to all women in the model, not just those with fear of childbirth. As described in a Swedish qualitative study, the presence of a known midwife and the common understanding developed through repeated interactions were highly valued during childbirth.^13^ Another recently published Swedish cohort study showed improved pregnancy outcomes in an MCoC model for women with fear of childbirth.^38^

In this study, 23.2% of the women experienced fear of childbirth, defined as a FOBS score of 60 or higher,^19^ which is similar to other studies.^20^ According to the Swedish Pregnancy Register, 13% of all pregnant women received counseling for fear of childbirth in 2023. The national average was 11% among primiparous women and 15% among multiparous women who received counseling for fear of childbirth. The arrangements included extra visits to midwives, physicians, psychologists, and/or sociologists, as well as additional time for therapeutic conversations with antenatal midwives.^14^ Although the counseling itself does not directly correspond to the number of women experiencing fear of childbirth, it indicates the phenomenon as common among women in Sweden.

### Satisfaction With Care

Interest in MCoC was also associated with satisfaction with care in all 3 dimensions: emotional, physical, and overall assessment. Women who were less satisfied or dissatisfied with their care showed more interest in MCoC, with the strongest association seen in the overall assessment of care. This can be interpreted as a desire for an alternative to the current care experience. As mentioned, MCoC is associated with more spontaneous vaginal births, fewer cesarean births, and fewer interventions,^2^ factors that women dissatisfied with their care might find appealing. The positive outcomes and higher satisfaction among women in MCoC^2^ contribute to the WHO's recommendations for a positive childbirth experience.^4^ This recommendation is intended for all women, indicating that MCoC could serve as a vehicle to improve satisfaction with care during pregnancy, childbirth, and postpartum.

Free‐text responses provided valuable insights that complemented the numerical results. When women used their own words to describe their thoughts on MCoC, it became evident that they sought reassurance about safety and security, with the word *safety* mentioned 220 times. According to the women's own words, MCoC would make them feel safe during pregnancy and childbirth. Previous studies have shown that women in Sweden feel safe and calm in an MCoC model.^13^, ^32^ In the present study, women expressed a desire to know their midwife and build a relationship over time, which is recognized as one of the most powerful aspects of the MCoC, model as described in a systematic review.^8^ Overall, women in this study associated MCoC with positive outcomes. The MCoC model was generally seen as a matter of course, as something they should not have had to request. This finding aligns with a scoping review of 59 Australian studies on women's interests and needs in maternity care, which concluded that continuity of care, particularly MCoC, was highly requested. Women expressed a strong desire for autonomy and involvement in decisions regarding their model of care, level of support, and interventions received. The feeling of safety was a prominent theme.^39^

Conversely, negative previous experiences of giving birth in a Swedish hospital and lack of confidence in the health care system contributed to women's choice of giving birth at home unassisted, also known as *free birth*. As these women were unable to get what they wished for from the national health care system, the only reasonable option for them was to give birth without a midwife.^40^, ^41^ In some cases, women would have preferred to give birth assisted by a midwife, but no home birth midwives were available.^41^ In the present study, one woman responded that she had chosen a free birth, and 26 women had planned home births assisted by a midwife. The relatively high proportion of home births (2.7%) in this sample indicates a notable interest in alternative birth settings and can be attributed to the participation of women in the established MCoC model in Sweden's capital, which includes the option of home birth. Offering MCoC to women in all parts of Sweden could empower them to have a birth experience in their own terms, regardless of the birthplace.

### Strengths and Limitations

The self‐selective nature of the questionnaire is a limitation, as women who are already interested in MCoC may be more likely to respond, leading to an overestimation of interest. However, 47% of the respondents had never heard of MCoC before being introduced to the concept through the study. In the comparison of the 2 groups, women who were familiar with the model expressed more interest than those who were unaware of it. This indicates that the limited information provided about MCoC did not convince all women that it was the best option. Conversely, this finding could also suggest that women who were interested in MCoC were more likely to respond to the questionnaire, which should be considered when interpreting the results. Analyses of women who did not wish to participate in the follow‐up questionnaire showed no differences regarding country of birth, partnership status, parity, or health care region. However, there were differences in education level and age groups. Women who were willing to participate were more likely to have a university or high school education and to belong to either the youngest or the oldest age groups.

When comparing study variables with the national sample of women from the Swedish Pregnancy Register, parity and birth mode were similar.^14^ The mean age was just slightly higher than the national average for pregnant women in Sweden (32.2 vs 31.4 years).^42^ This difference is understandable, as many of the participating women lived in the capital of Sweden, where pregnant women, in general, are older than in other parts of the country.^42^ Additionally, the education level in this sample was higher compared to the general female population.^43^ This limitation can be explained by the fact that the study did not target non‐Swedish‐speaking women. Excluding non‐Swedish‐speaking women is a weakness in the study. Due to time and financial constraints, the questionnaire was not translated into different languages. Further research should prioritize women with native languages other than Swedish.

To improve trustworthiness, the questionnaire was piloted both digitally and in hard copy, with only minor refinements made. The external validity was strengthened by the fact that the questionnaire was answered by women from all parts of Sweden. The objectivity was also enhanced by the digital format of the questionnaire. Additionally, the midwives who informed women about the study were not part of the research group and were not expected to influence the answers or the women's willingness to participate.

Approximately 30 women misspelled their email addresses, so the invitation to the follow‐up was not correctly delivered. In some cases, when it was obvious to the research team the letters were corrected (eg “.con” was changed to “.com” and “hitmail” was changed to “Hotmail”), but in some cases, it was impossible to decipher the incorrect characters and the follow‐up invitation was not received, explaining some of the dropout.

The longitudinal design is a strength, as women could assess their interest in MCoC both during pregnancy and after experiencing fragmented standard childbirth care. Enhancing the understanding of women's preferences could facilitate the design and implementation of MCoC in Sweden.

## CONCLUSION

Pregnant women and new mothers in Sweden show a high level of interest in MCoC. However, the current maternity services do not adequately meet these preferences, indicating the need to scale up MCoC, particularly for women experiencing fear of childbirth and those who are dissatisfied with their care. Maternity services and childbirth care in Sweden should be enhanced to better align with the needs and preferences of pregnant women and new mothers.

## CONFLICTS OF INTEREST

The authors have no conflicts of interest to disclose.

H.F. is supported by her employer Region Uppsala (LUL‐987136, LUL‐1001837). The funding did not influence the design or conduct of research.

## Supporting information




**Appendix** **S1**. Strengthening the Reporting of Observational Studies in Epidemiology (STROBE) Checklist.
